# *Mycobacterium avium* Infection Induces H-Ferritin Expression in Mouse Primary Macrophages by Activating Toll-Like Receptor 2

**DOI:** 10.1371/journal.pone.0082874

**Published:** 2013-12-09

**Authors:** Sandro Silva-Gomes, Cécile Bouton, Tânia Silva, Paolo Santambrogio, Pedro Rodrigues, Rui Appelberg, Maria Salomé Gomes

**Affiliations:** 1 IBMC, Instituto de Biologia Molecular e Celular, Universidade do Porto, Porto, Portugal; 2 ICBAS, Instituto de Ciências Biomédicas Abel Salazar, Universidade do Porto, Porto, Portugal; 3 Institut de Chimie des Substances Naturelles, UPR2301 CNRS, Centre de Recherche de Gif, Gif-sur-Yvette, France; 4 San Raffaele Scientific Institute, DIBIT, Milan, Italy; CINVESTAV-IPN, Mexico

## Abstract

Important for both host and pathogen survivals, iron is a key factor in determining the outcome of an infectious process. Iron with-holding, including sequestration inside tissue macrophages, is considered an important strategy to fight infection. However, for intra-macrophagic pathogens, such as *Mycobacterium avium*, host defence may depend on intracellular iron sequestration mechanisms. Ferritin, the major intracellular iron storage protein, plays a critical role in this process. In the current study, we studied ferritin expression in mouse bone marrow-derived macrophages upon infection with *M. avium*. We found that H-ferritin is selectively increased in infected macrophages, through an up-regulation of gene transcription. This increase was mediated by the engagement of Toll like receptor-2, and was independent of TNF-alpha or nitric oxide production. The formation of H-rich ferritin proteins and the consequent iron sequestration may be an important part of the panoply of antimicrobial mechanisms of macrophages.

## Introduction

As an essential nutrient for both host and invader, iron plays a central role in determining the outcome of infections. Infection leads to a re-distribution of iron in the body of vertebrate animals, so that less iron is found in circulation and more iron is sequestered inside macrophages [1]. While the decrease in circulating iron is a critical step for the control of the growth of extra-cellular microbes, it is less clear how the increased sequestration of iron inside macrophages impacts on intra-macrophagic infections. There are several microbes that reside and proliferate inside the macrophages of the animals they infect. These agents, including mycobacteria, leishmania, and salmonella, tend to cause chronic infections. Previous work by ours and other groups has shown that iron availability can clearly affect the growth of mycobacteria during experimental infection of mice. In iron-overloaded mice, mycobacteria grow more extensively, while they grow less in mice rendered iron deficient [2–5]. These results can be reproduced *in cellulo* using the model of infection of bone marrow-derived mouse macrophages, infected with *Mycobacterium avium* [2]. Given the clear evidence that iron availability affects the growth of *M. avium* inside macrophages, it is relevant to understand whether macrophages are able to modulate intracellular iron availability to the microbes they harbour. 

Ferritin is a key regulator of the intracellular iron metabolism through the storage of iron. In mammals, cytosolic ferritin is formed by 24 subunits of heavy (H) and light (L) chains, that spontaneously assemble into a shell-like structure, capable of storing up to 4500 iron atoms in its mineral core [6]. H-ferritin has a ferroxidase centre that promotes the conversion of Fe^2+^ to Fe^3+^, and L-ferritin facilitates nucleation and mineralization of the iron core [7]. Ferritin subunits are not interchangeable, as shown by the embryonic lethality of H-ferritin gene deletion [8]. H- and L-subunits are encoded by separate genes, which are differentially regulated [9]. As many other iron-related proteins, ferritin expression can be regulated post-transcriptionally by iron regulatory proteins (IRP), which interact with the *cis*-regulatory iron responsive elements (IRE) present in the 5’ UTR of both ferritin mRNAs [10]. 

In the present work, we investigated the regulation of ferritin in mouse bone marrow-derived macrophages infected with *M. avium*. We show that *M. avium* induces an increase of H-ferritin mRNA, and concomitant protein level, which is dependent on the engagement of Toll-like receptor-2, and independent of the production of TNF-alpha and endogenous nitric oxide. 

## Materials and Methods

### Animals

C57BL/6 mice were bred at IBMC. TLR2-deficient mice, on a C57BL/6 background [11] were bred at the IBMC from a breeding pair kindly provided by Dr Shizuo Akira. TNF-alpha-deficient mice, on a C57BL/6 background, were bred at the IBMC from breeders purchased from B&K Universal (East Yorkshire, United Kingdom). Inducible nitric oxide synthase (iNOS) – deficient mice [12], on a C57BL/6 background, were bred at the IBMC from a breeding pair kindly provided by Drs J. Mudgett, J.D. MacMicking and C. Nathan (Cornel University, New York). All mice were kept at the IBMC animal facility in high efficiency particulate air (HEPA)-filter-bearing cages and fed sterile chow and autoclaved water.

All animal maintenance and manipulations were conducted according to the rules of the IBMC animal ethics committee. This study was carried out in strict accordance with the recommendations of the European Convention for the Protection of Vertebrate Animals used for Experimental and Other Scientific Purposes (ETS 123) and 86/609/EEC Directive and Portuguese rules (DL 129/92). The animal experimental protocol was approved by the competent national authority Direcção Geral de Veterinária (DGV) (Protocol Permit Number: 0420/000/000/2011)

### Bacteria

*Mycobacterium avium* strain 2447, smooth transparent (SmT), was isolated from an AIDS patient and given to us by Dr. F. Portaels, Institute of Tropical Medicine, Antwerp, Belgium. The bacteria were grown in Middlebrook 7H9 Broth (Difco) with 0.05% Tween 80 (Sigma). Cultures were harvested during exponential phase, centrifuged, washed in saline with Tween 80, briefly sonicated and stored in aliquots at -80 °C until used.

### Macrophage culture

Bone marrow cells were flushed from mice femurs with ice cold Hank’s Balanced Salt Solution (HBSS, Gibco), collected by centrifugation and resuspended in Dulbecco’s Modified Eagle’s Medium (DMEM, Gibco) containing 10% Foetal Bovine Serum (FBS, Gibco) and 10% L929 Cell Conditioned Medium (LCCM), as a source of Macrophage-Colony Stimulating Factor (M-CSF). The cells were distributed in 24-well plates and incubated at 37 °C in a 7% CO2 atmosphere. Three days after seeding, another 0.1 ml LCCM was added. On the 7th day, the medium was renewed. On the 10th day of culture, when cells were completely differentiated into macrophages, some wells were infected with *M. avium*, by substituting the medium with 0.2 ml of DMEM containing 10^6^ CFU of *M. avium* (approximately 10 bacteria per macrophage). In uninfected controls, the medium was replaced with an equal volume of DMEM. Cells were incubated for 4h at 37°C in a CO_2_ atmosphere and then washed with warm HBSS to remove non-internalized bacteria and reincubated in DMEM, with 10% FBS and 10% LCCM. To block the transcription of the cell, some macrophages were incubated for 15 min with 1 µg/ml Actinomycin D (Sigma) in DMEM at 37 °C before infection. In some experiments, macrophages were incubated with 1 ng/ml of the synthetic diacylated lipoprotein FSL-1 (InvivoGen) in complete medium.

### Quantification of H- and L-Ferritin

Macrophages were washed with cold saline and lysed with 400 μl of a solution containing 50 mM Hepes (Gibco), 1% IGEPAL C-630 (Sigma) and 1% proteases inhibitor cocktail P840 (Sigma). Ferritin concentrations in the lysates were determined by ELISA assays using polyclonal antibodies (Abs) raised against mouse recombinant H- and L-ferritin subunits and calibrated with the corresponding recombinant homopolymers. The specificity and the absence of cross-reactivity of the Abs have been previously described [13]. Additional tests have been performed to confirm the absence of cross-reactivity with mycobacterial antigens. The results are expressed as ng of ferritin per mg of total protein in the cell lysate. Total protein content was measured using the BCA protein assay kit (Pierce).

### ^55^Fe uptake and incorporation into ferritin

BMM uninfected or infected with *M. avium* for 24h were incubated with 2.5 µM (^55^Fe) ferric ammonium citrate (Perkin Elmer) for 6h, at 37 °C in a 7% CO_2_ atmosphere. Afterwards, cells were washed with cold saline and lysed with 25 mM Tris-HCl buffer pH 7.4 (Sigma), containing 0.5% Triton X-100 (Sigma) and 1% proteases inhibitor cocktail P840 (Sigma). Total protein content was measured on soluble homogenates using Bio-Rad DC^TM^ Protein Assay (Bio-Rad) and 18 µg were loaded on a native PAGE (7.5% acrylamide, 1.5 mm thick). The gel was dried, subjected to phosphor imaging (Typhoon 8600; Molecular Diagnostics, Amersham Biosciences), and analyzed using the ImageQuant program version 5.1.

### Gene expression

Total RNA was extracted using the Micro-to-Midi Total RNA Purification System (Invitrogen) according to the manufacturer’s specifications. 2 µg of total RNA was transcribed into cDNA, by a Moloney Murine Leukemia Virus Reverse Transcriptase (Fermentas), using an oligo(dT)_18_ primer. The primers used for amplification of cDNA were as follows: *Hprt1* (housekeeping) 5’- gtaatgatcagtcaacgggggac -3’ (forward) and 5’-ccagcaagcttgcaaccttaacca-3’ (reverse); *Fth1* 5’- ggagttgtatgcctcctac -3’ (forward) and 5’- gagatattctgccatgcc -3’ (reverse). The primers were shown not to co-amplify genomic DNA. All reactions were performed in a total reaction volume of 20 µL with iQ™ SYBR^®^ Green Supermix (Bio-Rad) and carried out in the iQ™5 instrument (Bio-Rad). Baseline thresholds were calculated by Bio-Rad iQ5 program and the threshold cycles (Ct) were used in the REST software [14], where CT values for target gene were normalized to expression levels of *Hprt1*. Values are reported as n-fold difference relative to the control samples.

### Electromobility Shift Assay

BMM were washed with cold PBS and incubated for 10 min on ice with digitonin (Sigma) at 0.007% in sucrose 0.25M and Hepes 0.1M, pH 7.4, and centrifuged 10 min at 1800 g. The supernatant was centrifuged for 1h at 100 000 g and the mitochondria-free cytosolic extract was collected. The samples were kept at -80 °C until use. RNA-protein interactions were performed as described previously [15,16] using 4 μg of cytosolic extract and a molar excess of [alpha-^33^P]UTP- labeled H-ferritin IRE *in vitro* transcribed from plasmid pSPT-fer (kindly provided by Dr. L.C. Kühn, ISREC, Switzerland). IRE-IRP complexes were resolved in 6% nondenaturing polyacrylamide gel. Samples were treated in parallel with 2% 2-mercaptoethanol prior to the addition of the ^33^P-labeled IRE probe to fully activate IRP-IRE binding activity. 

### Statistical analysis

Data was analysed using a two-tailed unpaired student’s t test. 

## Results

### 1. *Mycobacterium avium* infection increases ferritin heavy chain levels in bone marrow-derived macrophages through transcriptional activation

**Figure 1:**
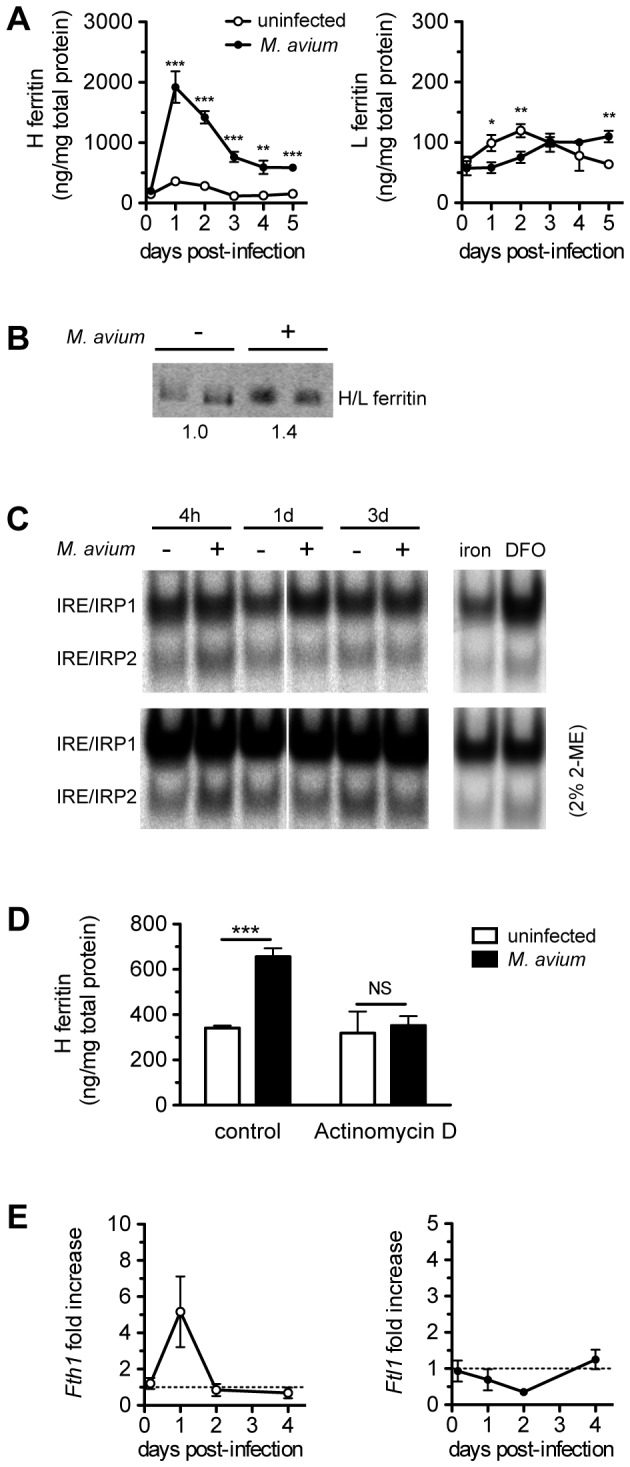
Effect of *Mycobacterium avium* infection on intramacrophagic ferritin. Bone marrow-derived macrophages were obtained from C57Bl/6 mice and infected with *M. avium*, as described in Material and Methods, or left uninfected. A - At different time points, macrophages were lysed and the amount of ferritin was quantified by ELISA. Data are presented as ng of ferritin per mg of total protein. The results are shown as average ± SD from one experiment performed in triplicate out of four independent experiments. Superscripts indicate statistical significance between M. avium-infected and uninfected, within the correspondent time-point, as follows: *p<0.05, **p<0.01, ***p<0.001. B – BMM uninfected or infected with *M. avium* for 24h were incubated for 6h with (^55^Fe) ferric ammonium citrate. Total protein (18 µg) was loaded (in duplicates) in native PAGE and exposed to autoradiography to analyze protein-bound iron. A single band was detected corresponding to cytosolic H/L ferritin. The values indicate the average relative band intensity for each condition. C – BMM infected with *M. avium* for 4h, 1 and 3 days and respective uninfected controls were tested for IRP-IRE binding activity, by gel retardation assay. 2% of 2-mercaptoethanol (2-ME) fully activates IRP binding activity and shows equal loading. BMM treated with iron or deferoxamine (DFO) were tested in a separated gel to confirm the reliability of the assay. D – BMM were treated with the transcriptional inhibitor actinomycin D or with vehicle. After an 8h-infection with *M. avium*, the BMM were lysed and H- and L-ferritin were quantified by ELISA. Results show the average + SD from one experiment performed in triplicate out of three independent experiments. ***p<0.001, NS not significant. E – At different time points, total RNA was collected from macrophages and the expression levels of ferritin genes was quantified by qRT-PCR, and normalized to *Hprt1*. Results are shown as fold increase in *M. avium*-infected macrophages in comparison with uninfected ones. Data are presented as average ± SE from one experiment performed in triplicate from a total of two independent experiments.

To test whether *M. avium* infection alters intracellular ferritin levels, we infected mouse bone marrow-derived macrophages (BMM) and analysed the ferritin protein levels for up to 5 days. The infection led to a 5.3±0.9 fold increase of ferritin heavy chain in the first 24h, which remained elevated until day 5 (3.8±0.4 fold increase) (Figure 1A and Table S1). As for ferritin light chain, we found it to be regulated to a lesser extent by the infection. L-ferritin levels tended to decrease in infected BMM by 24h, and to be restored at day 3 of infection (Figure 1A). 

We then tested if the increase in ferritin induced by infection leads to an increased diversion of iron into storage. BMM infected or not with *M. avium* were pulsed with ^55^Fe ferric ammonium citrate and iron incorporation in ferritin was analysed in a native PAGE followed by exposure to autoradiography film. As observed in Figure 1B, infected BMM had a 40% increase of ferritin- bound- ^55^Fe.

The expression of ferritins can be regulated post-transcriptionally by iron-regulatory proteins (IRPs). The binding of IRPs to the unique iron-responsive elements (IRE), which is present in the 5’ untranslated region of both L- and H-ferritin mRNAs, blocks the mRNA translation [10]. Therefore, a decreased binding capacity of IRP could contribute to H-ferritin protein increase. Cytosols from control and infected BMM were tested for their IRP-IRE binding activity during the course of the infection. Results in Figure 1C show that the IRE-IRP regulatory system is kept at a low basal activity in both control and infected BMM over the 3 days post-infection. Moreover, inhibiting the cell transcription with actinomycin D blocked the H-ferritin increase upon infection (Figure 1D), suggesting transcriptional, rather than post-transcriptional regulation by infection. To confirm this, we followed the expression of the genes coding for ferritin chains upon infection by qRT-PCR. *Fth1*, coding for H-ferritin, was found to be up-regulated by 5.2±1.9 fold in infected BMM at 24h (Figure 1E), correlating with the peak of protein expression (Figure 1A). The gene coding for L-ferritin (*Ftl1*), in accordance with the protein, was less regulated, with a reduction of 65% at day 2 of infection (Figure 1E).

### 2. Ferritin is regulated by infection independently of TNF-alpha or NO production

**Figure 2:**
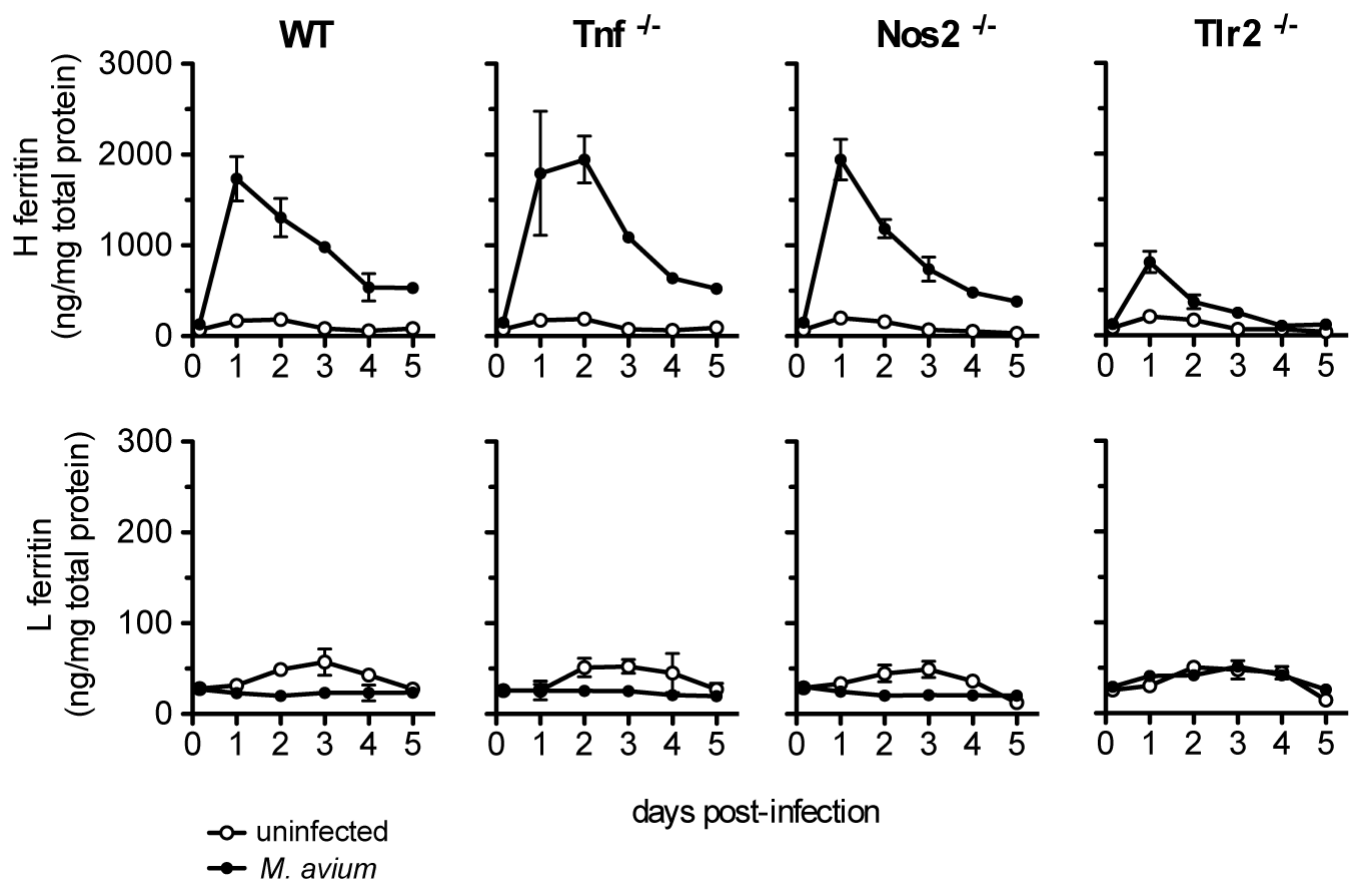
Effect of *M. avium* infection on ferritin content in the absence of TNF-alpha, iNOS and TLR-2. Bone marrow-derived macrophages were obtained from C57Bl/6 (WT), TLR-2^-/-^, TNF-alpha^-/-^ and NOS2^-/-^ mice. BMM were infected and the ferritin content was quantified as described in Figure 1. The results are shown as average ± SD from one experiment performed in triplicate out of two independent experiments.

The infection of BMM with mycobacteria induces TNF-alpha production [17], a cytokine that (specifically) induces the expression of H-ferritin by an increase in *Fth1* transcription [18]. To investigate if the increase in H-ferritin observed during *M. avium* infection is a consequence of TNF-alpha production, we infected BMM genetically deficient in the production of TNF-alpha and followed the ferritin expression. As can be seen in Figure 2, the absence of TNF-alpha did not hamper the regulation of ferritin by infection, indicating that the increase in H-ferritin is not mediated by the production of this cytokine.

The infection of BMM with various mycobacteria also induces nitric oxide (NO) production [19]. Moreover, it has been recently reported that BMM exposed to exogenous NO can transcriptionally up-regulate ferritin expression [20]. To determine whether production of endogenous NO contributes to ferritin regulation during infection, we used BMM deficient in the NO synthase2 (NOS2). As shown in Figure 2, NOS2^-/-^ BMM exhibited similar ferritin protein profile changes to those of WT BMM post infection, demonstrating that NO is not involved in the regulation of ferritin by *M. avium*.

### 3. Ferritin regulation by *M. avium* infection is dependent on the activation of Toll-Like Receptor 2

**Figure 3:**
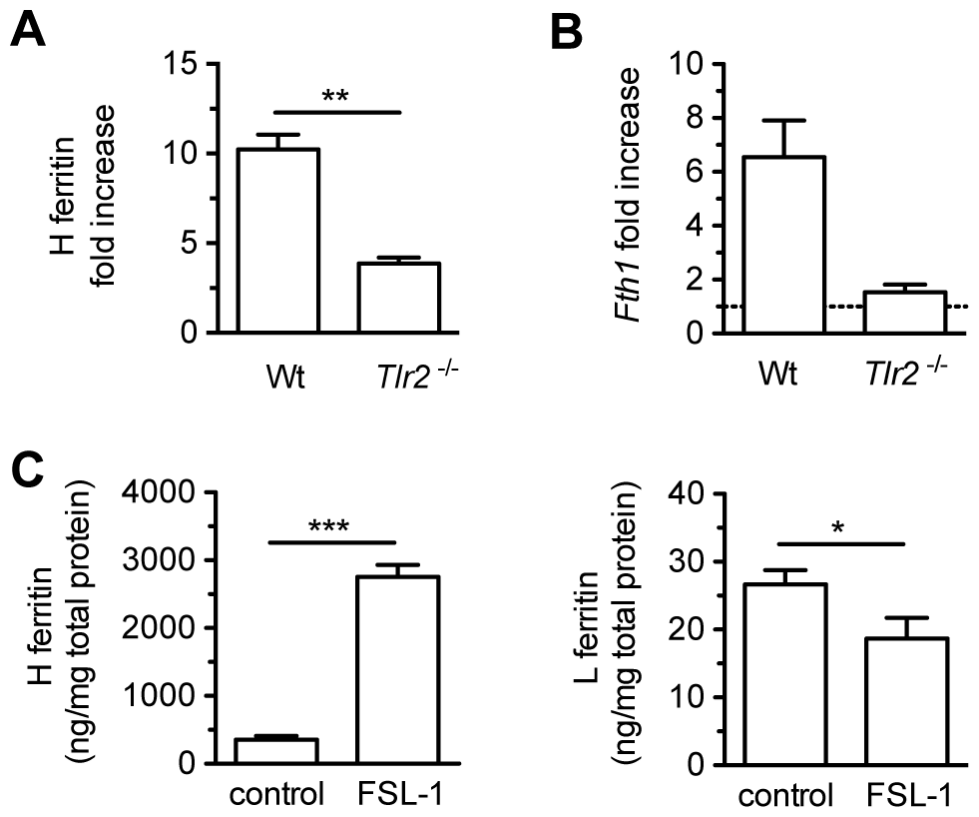
TLR-2 activation leads to increased expression of H-ferritin. A, B – BMM from C57Bl/6 (WT) and TLR-2^-/-^ mice were left uninfected or infected for 24h with *M. avium*. The H-ferritin fold increase in infected BMM in comparison with uninfected ones is shown at the protein level (A) and mRNA (B). C – BMM were treated with the TLR-2 agonist FSL-1 for 24h, and the levels of H- and L-ferritin was quantified by ELISA. Results show the average + SD from one experiment performed in triplicate out of three independent experiments. Statistical differences as described in Figure 1.

Toll-Like Receptor (TLR)-2 is the main receptor for the recognition of several mycobacterial constituents [21]. To evaluate the role of TLR-2 in the regulation of ferritin by the mycobacteria, we used TLR-2^-/-^ macrophages and measured the cell ferritin content during the infection. Although TLR-2^-/-^ BMM increased H-ferritin in response to infection, this effect was markedly reduced in comparison with WT BMM. The absence of TLR-2 reduced by 62% the increase of H-ferritin after 24h of infection (Figure 2 and Figure 3A) and as much as 83% after 4 days (Figure 2). In accordance, TLR-2^-/-^ BMM did not increase *Fth1* mRNA expression upon infection (Figure 3B). Furthermore, L-ferritin was not regulated by the infection in TLR-2^-/-^ BMM (Figure 2). It could be argued that the engagement of TLR-2 might activate a pathway necessary, but not sufficient, to induce H-ferritin expression. To investigate if the activation of TLR-2 signalling is able, *per se*, to induce the increase in H-ferritin observed in *M. avium* infection, we used the synthetic ligand FSL-1 to specifically activate TLR-2 [22]. The stimulation of BMM with FSL-1 had a similar effect to the infection with *M. avium* (Figure 3C), strongly inducing H-ferritin, while slightly reducing L-ferritin content. These results show that TLR-2 engagement is the main responsible for ferritin regulation during *M. avium* infection.

## Discussion

Ferritin plays a major role in the control of iron distribution, and is regulated by iron, cytokines, hormones, growth factors and oxidants [9]. In the present work, we show that H-ferritin is induced in mouse bone marrow derived macrophages infected with *M. avium*. Previous studies had suggested that infection with intracellular pathogens can modulate ferritin expression. However, most studies were performed with immortal cell lines and the results were frequently in conflict with each other [23–27]. 

Our results, obtained with primary mouse macrophages, showed that the infection with *M. avium* increases the H-ferritin cell content, by acting at the transcriptional level. Furthermore, we showed that the infection did not lead to any observable change in IRE/IRP binding activity, likely because macrophages infected with *M. avium* produce lower flux of nitric oxide (a well-known activator of the IRE/IRP regulatory system) than macrophages infected with non pathogenic species [19]. Accordingly, elevated H-ferritin content was maintained in NOS2^-/-^ BMM infected with *M. avium* similarly to that of WT infected BMM. Our results further show that activation of TLR-2 increases H-ferritin through the activation of gene transcription, independently of NO. A recent report demonstrated that LPS (a TLR4 ligand) in combination with IFN-gamma strongly induced H-ferritin in IRP1^-/-^ BMM via endogenous NO production while H-ferritin content was maintained low in WT BMM [20]. Therefore, TLR-2 and TLR-4, which recognize different pathogen associated molecular patterns, are likely to have the same effect on ferritin, but adopting different pathways. 

TLR-2 is the main receptor for the innate recognition of several mycobacterial constituents [21]. TLR-2 activation results in the killing of *M. tuberculosis* by NO-dependent mechanisms [28] and in bacteriostasis of *M. avium* [29] by mechanisms that remain elusive. Here, we found that TLR-2 engagement leads to H-ferritin increase, which may contribute to the anti-mycobacterial activity induced by TLR-2 engagement. Although we found that TLR-2 activation was sufficient for H-ferritin up-regulation, TLR-2^-/-^ BMM were still able to increase H-ferritin in response to *M. avium* infection, albeit to a much lesser extent. This suggests that other Pattern Recognition Receptors could have a minor role in the recognition of *M. avium* and consequent induction of H-ferritin.

*M. avium* infection had a much less pronounced effect on the levels of L-ferritin, overall resulting in the formation of ferritin proteins richer in H-chain. This may have important physiological consequences, since a higher H/L ratio has been shown to reduce the cell’s labile iron pool [30]. Indeed, we found that *M. avium*-infected macrophages have a higher capacity for the incorporation of iron into cytosolic ferritin. Our results suggest, therefore, that H-ferritin up-regulation during infection may contribute to limit iron availability to mycobacteria, taking part of the macrophage nutriprive mechanisms [31]. On the other hand, H-ferritin has also been implicated in protection against oxidative stress [32,33] and apoptosis [34]. We did not detect an increase in the levels of oxidative damage of macrophages upon infection with *M. avium* (Figure S1) and we can suggest that the observed increase in H-ferritin can contribute to the cytoprotection of the host cell. Further studies, including loss of function assays, are needed to obtain more definitive insights on the role of H-ferritin in the restriction of *M. avium* growth inside macrophages. 

At a more systemic level, ferritin induction by microbial stimuli is likely to impair iron recycling by diverting it into storage, thus contributing to the development of the anemia associated with chronic disease (ACD) [35]. Several factors are involved in the development of ACD, including cytokines and hepcidin [36–40], but we have recently demonstrated that the anaemia observed during experimental mouse infection with *M. avium* is independent of hepcidin [41]. 

In summary, this work shows that the recognition of *M. avium* by macrophage TLR-2 leads to the increase of the expression of H-ferritin, through transcriptional activation, by mechanisms that although not completely elucidated, are independent of TNF-alpha and NO. TLR2 mediated-up regulation of H-Ft in primary macrophages may be important in host protection against mycobacterial infections, by causing pathogen iron starvation and host cell protection and may also contribute to the development of ACD during mycobacterial infections. These findings can help better understand the tight regulation of host iron metabolism which occurs during the innate immune response to infections.

## Supporting Information

Figure S1 **Effect of *Mycobacterium avium* infection on the oxidation of peroxiredoxins (Prx).** Bone marrow-derived macrophages were obtained from C57Bl/6 mice and infected with *M. avium*, as described in Material and Methods, or left uninfected. To evaluate oxidative damage, macrophages were lysed after 1 or 3 days and PrxSO3, formed by the overoxidation of Prx, was detected by western blot. Uninfected macrophages exposed to 100 µM H_2_O_2_ for 20 min were used as a positive control.
(DOCX)

Table S1 **Effect of *Mycobacterium avium* infection on intramacrophagic ferritin.** Bone marrow-derived macrophages were obtained from C57Bl/6 mice and infected with *M. avium* or left uninfected. At different time points macrophages were lysed and the amount of ferritin was quantified by ELISA. Day 0 refers to the time point immediately after infection. Data are presented as ng of ferritin per mg of total protein. The results are shown as average ± SD from one experiment performed in triplicate out of four independent experiments.
(DOCX)

## References

[1] Weinberg ED (1993) The development of awareness of iron-withholding defense. Perspect Biol Med 36: 215-221. PubMed: 8446492. 8446492 10.1353/pbm.1993.0063

[2] Gomes MS, Boelaert JR, Appelberg R (2001) Role of iron in experimental Mycobacterium avium infection. J Clin Virol 20: 117-122. doi:10.1016/S1386-6532(00)00135-9. PubMed: 11166658. 11166658

[3] Lounis N, Truffot-Pernot C, Grosset J, Gordeuk VR, Boelaert JR (2001) Iron and Mycobacterium tuberculosis infection. J Clin Virol 20: 123-126. doi:10.1016/S1386-6532(00)00136-0. PubMed: 11166659. 11166659

[4] Schaible UE, Collins HL, Priem F, Kaufmann SH (2002) Correction of the iron overload defect in beta-2-microglobulin knockout mice by lactoferrin abolishes their increased susceptibility to tuberculosis. J Exp Med 196: 1507-1513. doi:10.1084/jem.20020897. PubMed: 12461085. 12461085 PMC2194267

[5] Gomes-Pereira S, Rodrigues PN, Appelberg R, Gomes MS (2008) Increased susceptibility to Mycobacterium avium in hemochromatosis protein HFE-deficient mice. Infect Immun 76: 4713-4719. doi:10.1128/IAI.00612-08. PubMed: 18694968. 18694968 PMC2546818

[6] Harrison PM, Arosio P (1996) The ferritins: molecular properties, iron storage function and cellular regulation. Biochim Biophys Acta 1275: 161-203. doi:10.1016/0005-2728(96)00022-9. PubMed: 8695634. 8695634

[7] Arosio P, Levi S (2010) Cytosolic and mitochondrial ferritins in the regulation of cellular iron homeostasis and oxidative damage. Biochim Biophys Acta 1800: 783-792. doi:10.1016/j.bbagen.2010.02.005. PubMed: 20176086. 20176086

[8] Ferreira C, Bucchini D, Martin ME, Levi S, Arosio P et al. (2000) Early embryonic lethality of H ferritin gene deletion in mice. J Biol Chem 275: 3021-3024. doi:10.1074/jbc.275.5.3021. PubMed: 10652280. 10652280

[9] Torti FM, Torti SV (2002) Regulation of ferritin genes and protein. Blood 99: 3505-3516. doi:10.1182/blood.V99.10.3505. PubMed: 11986201. 11986201

[10] Muckenthaler MU, Galy B, Hentze MW (2008) Systemic iron homeostasis and the iron-responsive element/iron-regulatory protein (IRE/IRP) regulatory network. Annu Rev Nutr 28: 197-213. doi:10.1146/annurev.nutr.28.061807.155521. PubMed: 18489257. 18489257

[11] Takeuchi O, Hoshino K, Kawai T, Sanjo H, Takada H et al. (1999) Differential roles of TLR2 and TLR4 in recognition of gram-negative and gram-positive bacterial cell wall components. Immunity 11: 443-451. doi:10.1016/S1074-7613(00)80119-3. PubMed: 10549626. 10549626

[12] MacMicking JD, Nathan C, Hom G, Chartrain N, Fletcher DS et al. (1995) Altered responses to bacterial infection and endotoxic shock in mice lacking inducible nitric oxide synthase. Cell 81: 641-650. doi:10.1016/0092-8674(95)90085-3. PubMed: 7538909. 7538909

[13] Santambrogio P, Cozzi A, Levi S, Rovida E, Magni F et al. (2000) Functional and immunological analysis of recombinant mouse H- and L-ferritins from Escherichia coli. Protein Expr Purif 19: 212-218. doi:10.1006/prep.2000.1212. PubMed: 10833409. 10833409

[14] Pfaffl MW, Horgan GW, Dempfle L (2002) Relative expression software tool (REST) for group-wise comparison and statistical analysis of relative expression results in real-time PCR. Nucleic Acids Res 30: e36. doi:10.1093/nar/30.9.e36. PubMed: 11972351. 11972351 PMC113859

[15] Leibold EA, Munro HN (1988) Cytoplasmic protein binds in vitro to a highly conserved sequence in the 5' untranslated region of ferritin heavy- and light-subunit mRNAs. Proc Natl Acad Sci U S A 85: 2171-2175. doi:10.1073/pnas.85.7.2171. PubMed: 3127826. 3127826 PMC279951

[16] Müllner EW, Neupert B, Kühn LC (1989) A specific mRNA binding factor regulates the iron-dependent stability of cytoplasmic transferrin receptor mRNA. Cell 58: 373-382. doi:10.1016/0092-8674(89)90851-9. PubMed: 2752428. 2752428

[17] Sarmento AM, Appelberg R (1995) Relationship between virulence of Mycobacterium avium strains and induction of tumor necrosis factor alpha production in infected mice and in in vitro-cultured mouse macrophages. Infect Immun 63: 3759-3764. PubMed: 7558277. 7558277 10.1128/iai.63.10.3759-3764.1995 PMC173528

[18] Kwak EL, Larochelle DA, Beaumont C, Torti SV, Torti FM (1995) Role for NF-kappa B in the regulation of ferritin H by tumor necrosis factor-alpha. J Biol Chem 270: 15285-15293. doi:10.1074/jbc.270.25.15285. PubMed: 7797515. 7797515

[19] Roach SK, Schorey JS (2002) Differential regulation of the mitogen-activated protein kinases by pathogenic and nonpathogenic mycobacteria. Infect Immun 70: 3040-3052. doi:10.1128/IAI.70.6.3040-3052.2002. PubMed: 12010996. 12010996 PMC128028

[20] Styś A, Galy B, Starzyński RR, Smuda E, Drapier JC et al. (2011) Iron regulatory protein 1 outcompetes iron regulatory protein 2 in regulating cellular iron homeostasis in response to nitric oxide. J Biol Chem, 286: 22846–54. PubMed: 21566147. 21566147 10.1074/jbc.M111.231902 PMC3123052

[21] Means TK, Wang S, Lien E, Yoshimura A, Golenbock DT et al. (1999) Human toll-like receptors mediate cellular activation by Mycobacterium tuberculosis. J Immunol 163: 3920-3927. PubMed: 10490993. 10490993

[22] Mae M, Iyori M, Yasuda M, Shamsul HM, Kataoka H et al. (2007) The diacylated lipopeptide FSL-1 enhances phagocytosis of bacteria by macrophages through a Toll-like receptor 2-mediated signalling pathway. FEMS Immunol Med Microbiol 49: 398-409. doi:10.1111/j.1574-695X.2007.00218.x. PubMed: 17316370. 17316370

[23] Basaraba RJ, Bielefeldt-Ohmann H, Eschelbach EK, Reisenhauer C, Tolnay AE et al. (2008) Increased expression of host iron-binding proteins precedes iron accumulation and calcification of primary lung lesions in experimental tuberculosis in the guinea pig. Tuberculosis (Edinb) 88: 69-79. doi:10.1016/j.tube.2007.09.002. PubMed: 17942369. 17942369 PMC2271031

[24] McGarvey JA, Wagner D, Bermudez LE (2004) Differential gene expression in mononuclear phagocytes infected with pathogenic and non-pathogenic mycobacteria. Clin Exp Immunol 136: 490-500. doi:10.1111/j.1365-2249.2004.02490.x. PubMed: 15147351. 15147351 PMC1809054

[25] Carlyon JA, Ryan D, Archer K, Fikrig E (2005) Effects of Anaplasma phagocytophilum on host cell ferritin mRNA and protein levels. Infect Immun 73: 7629-7636. doi:10.1128/IAI.73.11.7629-7636.2005. PubMed: 16239567. 16239567 PMC1273867

[26] Nairz M, Theurl I, Ludwiczek S, Theurl M, Mair SM et al. (2007) The co-ordinated regulation of iron homeostasis in murine macrophages limits the availability of iron for intracellular Salmonella typhimurium. Cell Microbiol 9: 2126-2140. doi:10.1111/j.1462-5822.2007.00942.x. PubMed: 17466014. 17466014

[27] Pan X, Tamilselvam B, Hansen EJ, Daefler S (2010) Modulation of iron homeostasis in macrophages by bacterial intracellular pathogens. BMC Microbiol 10: 64. doi:10.1186/1471-2180-10-64. PubMed: 20184753. 20184753 PMC2838877

[28] Thoma-Uszynski S, Stenger S, Takeuchi O, Ochoa MT, Engele M et al. (2001) Induction of direct antimicrobial activity through mammalian toll-like receptors. Science 291: 1544-1547. doi:10.1126/science.291.5508.1544. PubMed: 11222859. 11222859

[29] Gomes MS, Sousa Fernandes S, Cordeiro JV, Silva Gomes S, Vieira A et al. (2008) Engagement of Toll-like receptor 2 in mouse macrophages infected with Mycobacterium avium induces non-oxidative and TNF-independent anti-mycobacterial activity. Eur J Immunol 38: 2180-2189. doi:10.1002/eji.200737954. PubMed: 18624355. 18624355

[30] Picard V, Epsztejn S, Santambrogio P, Cabantchik ZI, Beaumont C (1998) Role of ferritin in the control of the labile iron pool in murine erythroleukemia cells. J Biol Chem 273: 15382-15386. doi:10.1074/jbc.273.25.15382. PubMed: 9624120. 9624120

[31] Appelberg R (2006) Macrophage nutriprive antimicrobial mechanisms. J Leukoc Biol 79: 1117-1128. doi:10.1189/jlb.0206079. PubMed: 16603587. 16603587

[32] Cozzi A, Corsi B, Levi S, Santambrogio P, Biasiotto G et al. (2004) Analysis of the biologic functions of H- and L-ferritins in HeLa cells by transfection with siRNAs and cDNAs: evidence for a proliferative role of L-ferritin. Blood 103: 2377-2383. doi:10.1182/blood-2003-06-1842. PubMed: 14615379. 14615379

[33] Kakhlon O, Gruenbaum Y, Cabantchik ZI (2001) Repression of ferritin expression increases the labile iron pool, oxidative stress, and short-term growth of human erythroleukemia cells. Blood 97: 2863-2871. doi:10.1182/blood.V97.9.2863. PubMed: 11313282. 11313282

[34] Pham CG, Bubici C, Zazzeroni F, Papa S, Jones J et al. (2004) Ferritin heavy chain upregulation by NF-kappaB inhibits TNFalpha-induced apoptosis by suppressing reactive oxygen species. Cell 119: 529-542. doi:10.1016/j.cell.2004.10.017. PubMed: 15537542. 15537542

[35] Weiss G (2009) Iron metabolism in the anemia of chronic disease. Biochim Biophys Acta 1790: 682-693. doi:10.1016/j.bbagen.2008.08.006. PubMed: 18786614. 18786614

[36] Weiss G (2005) Modification of iron regulation by the inflammatory response. Best Pract Res Clin Haematol 18: 183-201. PubMed: 15737884. 15737884 10.1016/j.beha.2004.09.001

[37] Andrews NC (2004) Anemia of inflammation: the cytokine-hepcidin link. J Clin Invest 113: 1251-1253. doi:10.1172/JCI21441. PubMed: 15124013. 15124013 PMC398435

[38] Kim S, Ponka P (2002) Nitrogen monoxide-mediated control of ferritin synthesis: implications for macrophage iron homeostasis. Proc Natl Acad Sci U S A 99: 12214-12219. doi:10.1073/pnas.192316099. PubMed: 12209009. 12209009 PMC129424

[39] Alvarez-Hernández X, Licéaga J, McKay IC, Brock JH (1989) Induction of hypoferremia and modulation of macrophage iron metabolism by tumor necrosis factor. Lab Invest 61: 319-322. PubMed: 2788773. 2788773

[40] Laftah AH, Sharma N, Brookes MJ, McKie AT, Simpson RJ et al. (2006) Tumour necrosis factor alpha causes hypoferraemia and reduced intestinal iron absorption in mice. Biochem J 397: 61-67. doi:10.1042/BJ20060215. PubMed: 16566752. 16566752 PMC1479761

[41] Rodrigues PN, Gomes SS, Neves JV, Gomes-Pereira S, Correia-Neves M et al. (2011) Mycobacteria-induced anaemia revisited: A molecular approach reveals the involvement of NRAMP1 and lipocalin-2, but not of hepcidin. Immunobiology 216: 1127-1134. doi:10.1016/j.imbio.2011.04.004. PubMed: 21601942. 21601942

