# Supplementary material for: Mycobacterium avium Infection Induces H-Ferritin Expression in Mouse Primary Macrophages by Activating Toll-Like Receptor 2
Source: PLoS One. 2013 Dec 9;8(12):e82874. doi: 10.1371/journal.pone.0082874 (PMC3857292; doi:10.1371/journal.pone.0082874)
Supplement: Figure S1 — Effect of Mycobacterium avium infection on the oxidation of peroxiredoxins (Prx). Bone marrow-derived macrophages were obtained from C57Bl/6 mice and infected with M. avium, as described in Material and Methods, or left uninfected. To evaluate oxidative damage, macrophages were lysed after 1 or 3 days and PrxSO3, formed by the overoxidation of Prx, was detected by western blot. Uninfected macrophages exposed to 100 µM H2O2 for 20 min were used as a positive control. (DOCX) [file pone.0082874.s001.docx]

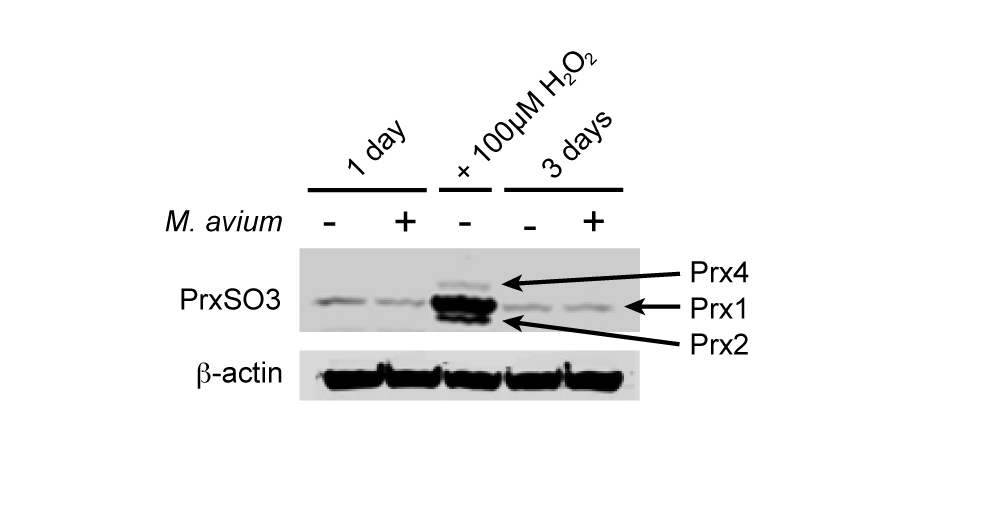


**Figure S1** - Effect of *Mycobacterium avium* infection on the oxidation of peroxiredoxins (Prx). Bone marrow-derived macrophages were obtained from C57Bl/6 mice and infected with *M. avium*, as described in Material and Methods, or left uninfected. To evaluate oxidative damage, macrophages were lysed after 1 or 3 days and PrxSO3, formed by the overoxidation of Prx, was detected by western blot. Uninfected macrophages exposed to 100 µM H_2_O_2_ for 20 min were used as a positive control.
