# Supplementary material for: Mycobacterium avium Infection Induces H-Ferritin Expression in Mouse Primary Macrophages by Activating Toll-Like Receptor 2
Source: PLoS One. 2013 Dec 9;8(12):e82874. doi: 10.1371/journal.pone.0082874 (PMC3857292; doi:10.1371/journal.pone.0082874)
Supplement: Table S1 — Effect of Mycobacterium avium infection on intramacrophagic ferritin. Bone marrow-derived macrophages were obtained from C57Bl/6 mice and infected with M. avium or left uninfected. At different time points macrophages were lysed and the amount of ferritin was quantified by ELISA. Day 0 refers to the time point immediately after infection. Data are presented as ng of ferritin per mg of total protein. The results are shown as average ± SD from one experiment performed in triplicate out of four independent experiments. (DOCX) [file pone.0082874.s002.docx]

**Table S1:** Effect of *Mycobacterium avium* infection on intramacrophagic ferritin.

|  | **H ferritin** | | | **L ferritin** | | |
| --- | --- | --- | --- | --- | --- | --- |
| **t (days)** | **uninfected** | ***M. avium*** | **Ratio** | **uninfected** | ***M. avium*** | **Ratio** |
| 0 | 147±31 | 201±43 | 1.4±0.4 | 69±7 | 57±12 | 0.8±0.2 |
| 1 | 360±41 | 1920±260 | 5.3±0.9 | 99±13 | 58±9 | 0.6±0.1 |
| 2 | 282±54 | 1419±102 | 5.0±1.0 | 120±11 | 76±9 | 0.6±0.1 |
| 3 | 117±28 | 764±85 | 6.5±1.7 | 100±15 | 101±8 | 1.0±0.2 |
| 4 | 125±58 | 591±110 | 4.7±2.3 | 78±25 | 100±4 | 1.3±0.4 |
| 5 | 152±15 | 586±23 | 3.8±0.4 | 64±3 | 110±10 | 1.7±0.2 |

Bone marrow-derived macrophages were obtained from C57Bl/6 mice and infected with *M. avium* or left uninfected. At different time points macrophages were lysed and the amount of ferritin was quantified by ELISA. Day 0 refers to the time point immediately after infection. Data are presented as ng of ferritin per mg of total protein. The results are shown as average ± SD from one experiment performed in triplicate out of four independent experiments.
